# Supplementary material for: Spatiotemporal proteomic profiling of the pro-inflammatory response to lipopolysaccharide in the THP-1 human leukaemia cell line
Source: Nat Commun. 2021 Oct 1;12:5773. doi: 10.1038/s41467-021-26000-9 (PMC8486773; doi:10.1038/s41467-021-26000-9)
Supplement: Supplementary file 1 — Supplementary Information [file 41467_2021_26000_MOESM1_ESM.pdf]

Supplementary Information

Spatiotemporal proteomic profiling of the dynamic pro-inflammatory response to lipopolysaccharide in the THP-1 human leukaemia cell line

Claire M. Mulvey[1,2#], Lisa M. Breckels[1#], Oliver M. Crook[1,3], David J. Sanders[4], Andre L.R. Ribeiro[4], Aikaterini Geladaki[1], Andy Christoforou[5], Nina Kočevar Britovšek[1,6], Tracey Hurrell[1], Michael J. Deery[1], Laurent Gatto[1,7], Andrew M. Smith[4, *] and Kathryn S. Lilley[1, *]

1. Cambridge Centre for Proteomics, Cambridge Systems Biology Centre and Department of Biochemistry, University of Cambridge, CB2 1QR, UK.

2. Present address: Cancer Research UK Cambridge Institute, University of Cambridge, Li Ka Shing Centre, Cambridge CB2 0RE, UK.

3. MRC Biostatistics Unit, Cambridge Institute for Public Health, Forvie Site, Robinson Way, Cambridge, CB2 0SR, UK.

4. Department of Microbial Diseases, Eastman Dental Institute, University College London, Royal Free Campus, Rowland Hill Street, London, NW3 2PF, UK.

5. Bristol Myers Squibb, 10300 Campus Point Drive, San Diego CA, USA.

6. Present address: Lek d.d., Kolodvorska 27, 1234 Mengeš, Slovenia.

7. Present address: de Duve Institute, UCLouvain, Avenue Hippocrate 75, Brussels, 1200, Belgium.

# these authors contributed equally

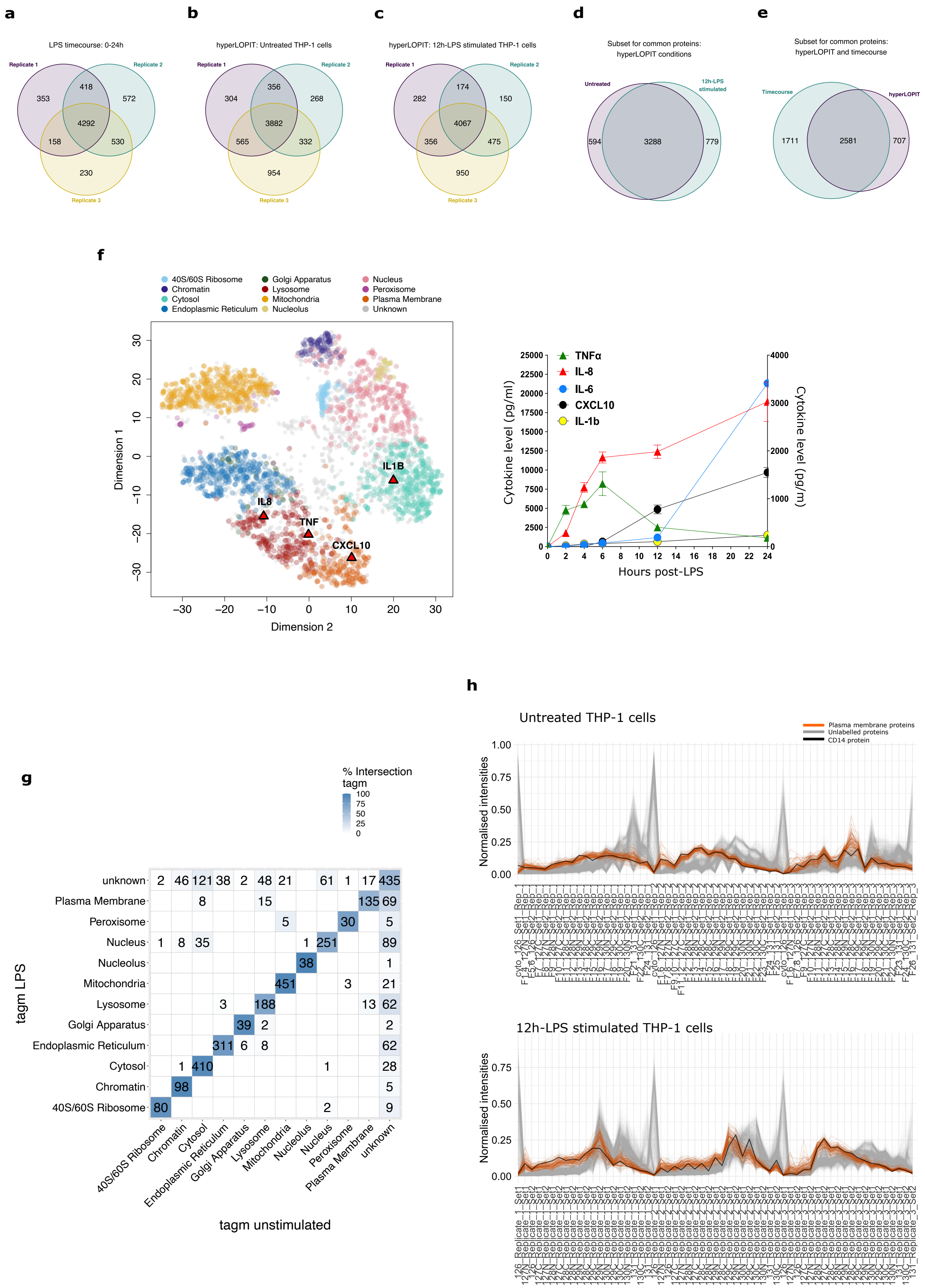
a
LPS timecourse: 0-24h
Replicate 1
Replicate 2
Replicate 3
353
418
572
4292
158
530
230
b
hyperLOPIT: Untreated THP-1 cells
Replicate 1
Replicate 2
Replicate 3
304
356
268
3882
565
332
954
c
hyperLOPIT: 12h-LPS stimulated THP-1 cells
Replicate 1
Replicate 2
Replicate 3
282
174
150
4067
356
475
950
d
Subset for common proteins: hyperLOPIT conditions
Untreated
12h-LPS stimulated
594
3288
779
e
Subset for common proteins: hyperLOPIT and timecourse
Timecourse
hyperLOPIT
1711
2581
707
f
40S/60S Ribosome
Chromatin
Cytosol
Endoplasmic Reticulum
Golgi Apparatus
Lysosome
Mitochondria
Nucleolus
Nucleus
Peroxisome
Plasma Membrane
Unknown
IL1B
IL8
TNF
CXCL10
Dimension 1
Dimension 2
TNFα
IL-8
IL-6
CXCL10
IL-1b
Cytokine level (pg/ml)
Cytokine level (pg/ml)
Hours post-LPS
g
% Intersection tagm
100
75
50
25
0
tagm LPS
tagm unstimulated
unknown
Plasma Membrane
Peroxisome
Nucleus
Nucleolus
Mitochondria
Lysosome
Golgi Apparatus
Endoplasmic Reticulum
Cytosol
Chromatin
40S/60S Ribosome
h
Untreated THP-1 cells
Plasma membrane proteins
Unlabelled proteins
CD14 protein
Normalised intensities
12h-LPS stimulated THP-1 cells
Normalised intensities

Supplementary Figure 1. Data overview showing the number of proteins quantified between the different LOPIT and time course triplicated experiments. **a** Venn diagram of the three biological replicate experiments for the 24 h LPS time-course experiment, showing that 4,292 proteins were quantified in all 3 replicates. **b** Venn diagram of the three biological replicate experiments for the unstimulated (0h-LPS) hyperLOPIT experiment, showing that 3,882 proteins were quantified in all 3 replicates. **c** Venn diagram of the three biological replicate experiments for the stimulated (12h-LPS) hyperLOPIT experiment, showing that 4,067 proteins were quantified in all 3 replicates. **d** The subset of 3,288 proteins which were identified in all 3 unstimulated (0h-LPS) and 3 stimulated (12h-LPS) hyperLOPIT datasets. **e** The subset of proteins which were identified in both the 24h-LPS time-course and in the hyperLOPIT analysis, providing temporal and spatial data for 2,581 proteins. **f** Four cytokine proteins (IL1B, CXCL10, TNF, IL8) were imputed from incomplete TMT profiles and overlaid onto the LPS-stimulated hyperLOPIT plot, to give an indication of their intracellular localisation. Secretory analysis of five cytokines (TNFα, IL8, IL6, CXCL10, IL1B) was determined by ELISA and demonstrated that the secretory process for each cytokine occurs at different time-frames following LPS stimulation. Cytokines represented as triangles (TNFα, IL8) are shown on the left Y-axis, whereas cytokines represented as circles (IL6, CXCL10, IL1B) are shown on the right Y-axis. *n* = 3 biologically independent experiments were conducted and data is plotted as the mean +/- *SD*. (G) Heatmap of overlapping organelle assignments from the TAGM classifications for unstimulated and LPS-stimulated datasets. (H) Protein abundance profile for CD14 (black) and for plasma membrane protein markers (orange) across all replicates in each condition for the hyperLOPIT experiments. All other proteins are shown as grey lines.

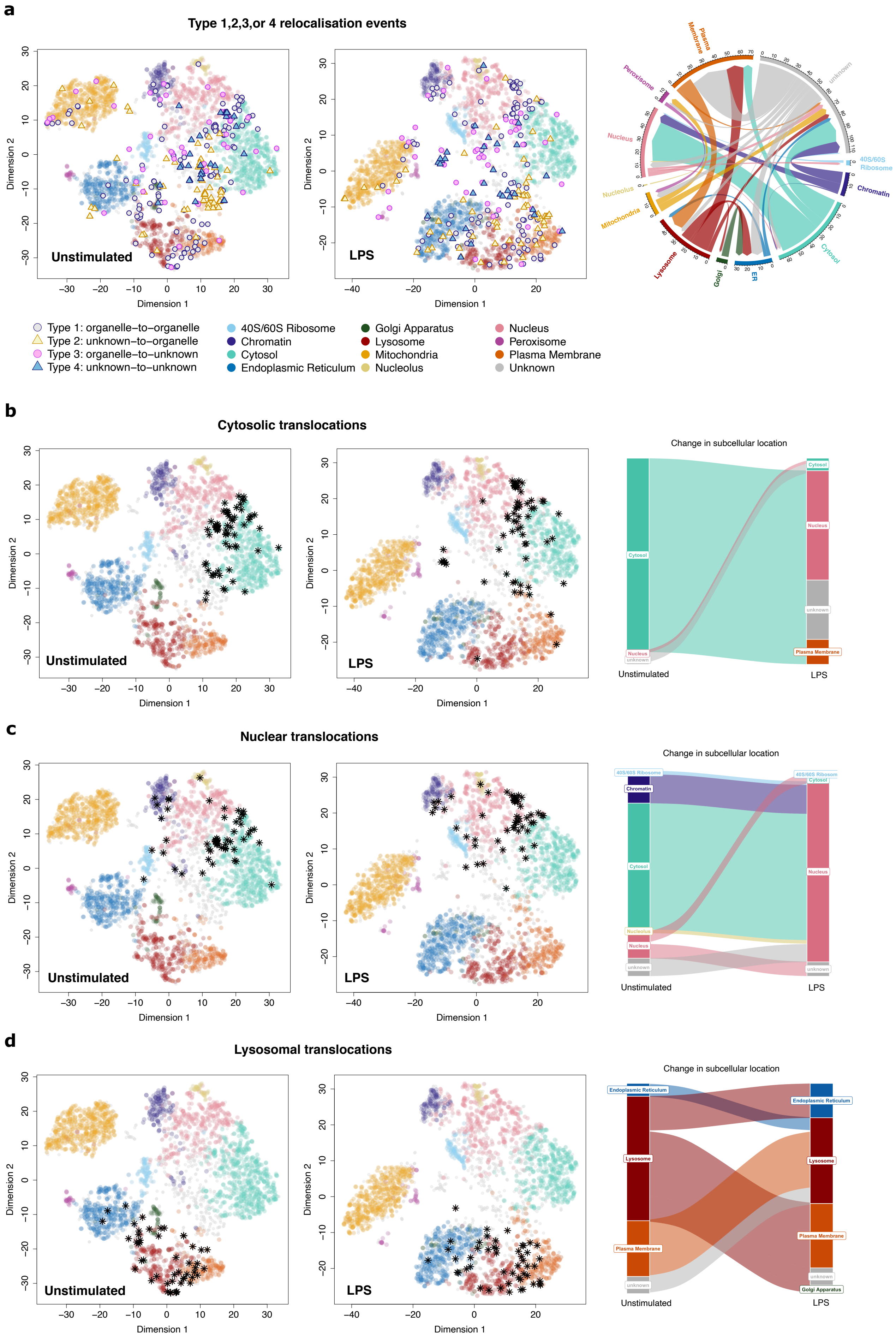
a
Type 1,2,3,or 4 relocalisation events
Unstimulated
LPS
Dimension 1
Dimension 2
Plasma Membrane
Peroxisome
Nucleus
Nucleolus
Mitochondria
Lysosome
Golgi
ER
Cytosol
Chromatin
40S/60S Ribosome
unknown
Type 1: organelle–to–organelle
Type 2: unknown–to–organelle
Type 3: organelle–to–unknown
Type 4: unknown–to–unknown
40S/60S Ribosome
Chromatin
Cytosol
Endoplasmic Reticulum
Golgi Apparatus
Lysosome
Mitochondria
Nucleolus
Nucleus
Peroxisome
Plasma Membrane
Unknown
b
Cytosolic translocations
Change in subcellular location
Cytosol
Nucleus
unknown
Plasma Membrane
Unstimulated
LPS
c
Nuclear translocations
Change in subcellular location
40S/60S Ribosome
Chromatin
Cytosol
Nucleolus
Nucleus
unknown
Unstimulated
LPS
d
Lysosomal translocations
Change in subcellular location
Endoplasmic Reticulum
Lysosome
Plasma Membrane
unknown
Golgi Apparatus
Unstimulated
LPS

Figure 2. Overview of translocation events. **a** Left/middle panel: t-SNE map showing the 253 relocalising proteins from the hyperLOPIT experiments and plotted by Type 1,2,3, or 4 relocalisation events, which are represented by grey circles, yellow triangles, pink circles and blue triangles, respectively. Right panel: Chord diagram (Circos plot) depicting the flow of all translocating proteins between different sub cellular compartments. Arrows show the direction of flow and are colour coded by organelle as per the legend and accompanying t-SNE plots. **b** Left/middle: t-SNE maps one each condition showing the 67 proteins which were found to translocate to/from the cytosol cluster. Right: alluvial plot summarising the flow of cytosolic translocations **c** Shown are 57 proteins which were found to translocate to/from the nucleus cluster. **d** Shown are 49 proteins which were found to translocate to/from the lysosome cluster.

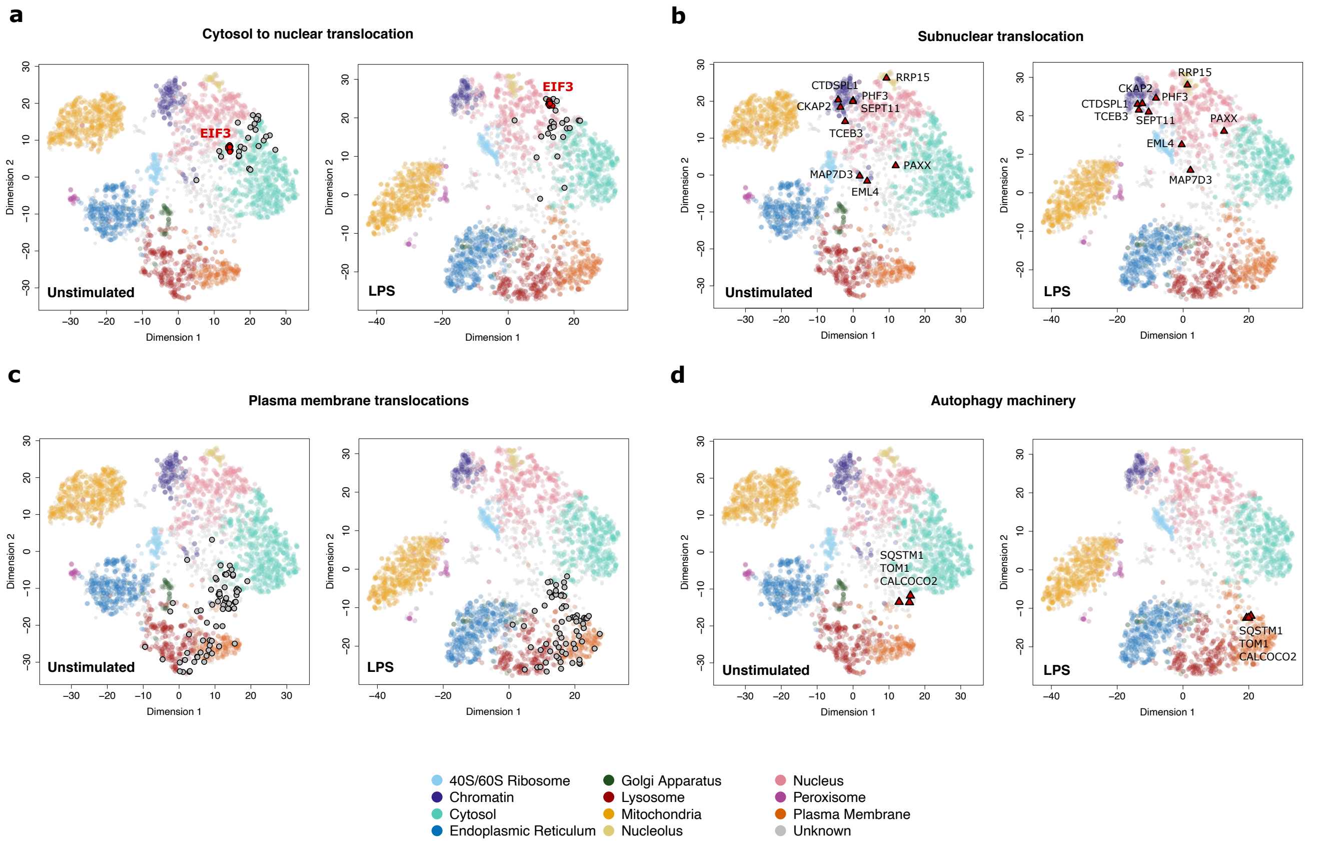

Supplementary Figure 3. Summary of nucleo-cytoplasmic and plasma membrane translocation events. **a** Shown are 37 proteins which were found to translocate between the cytosol and the nuclear clusters. **b** Shown are 9 proteins found to translocate between subnuclear compartments. **c** Shown are 70 proteins found to translocate to or from the PM. **d** Shown are three proteins known to co-localise: SQSTM1, CALCOCO2, TOM1.

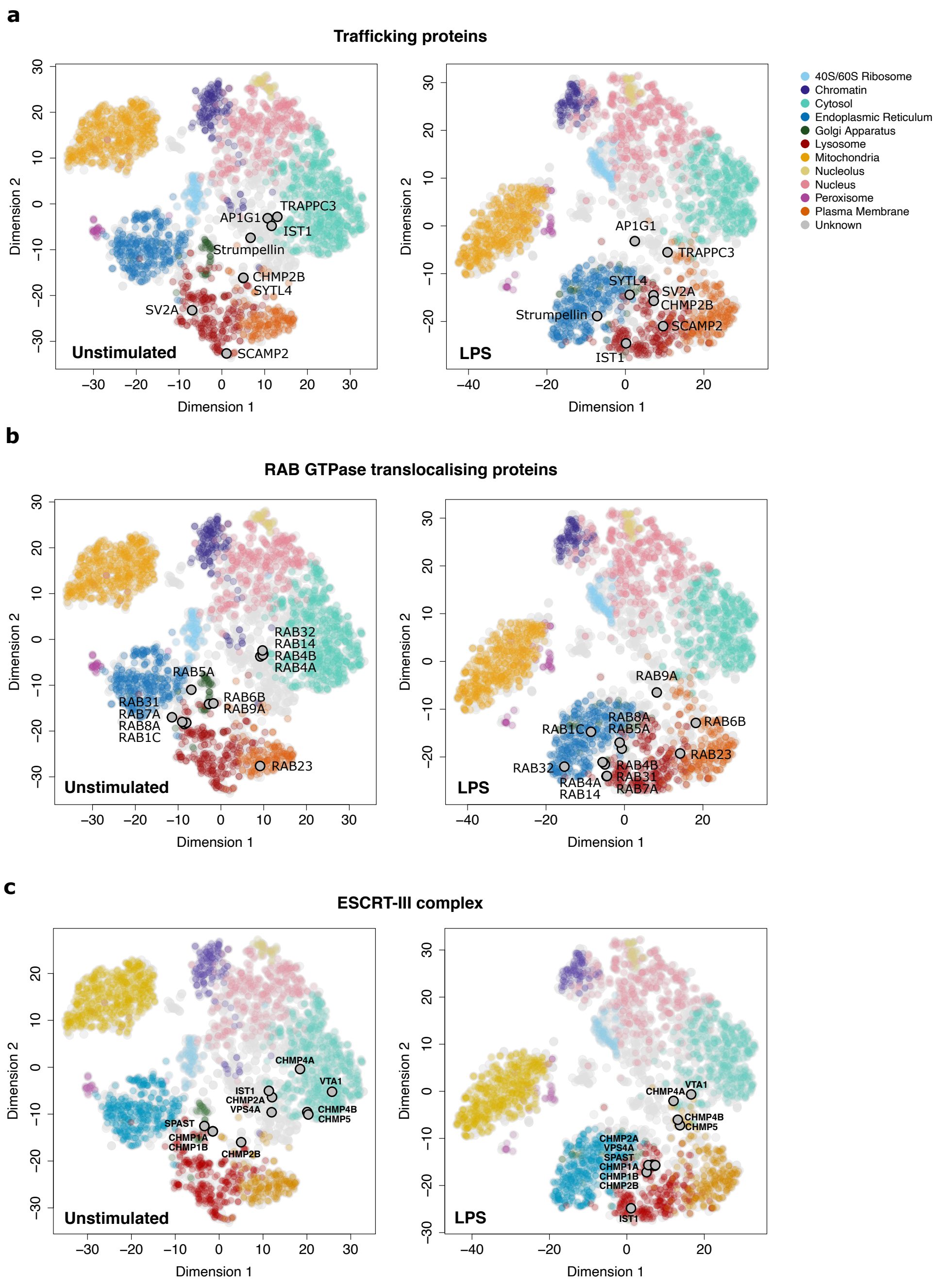

Supplementary Figure 4. T-SNE plots of translocating proteins involved in trafficking. **a** Shown are 8 trafficking proteins which were found to translocate following LPS stimulation. **b** Shown are 12 RAB GTPase trafficking proteins which were found to translocate following LPS stimulation. **c** Shown are 11 members of the ESCRT-III complex.

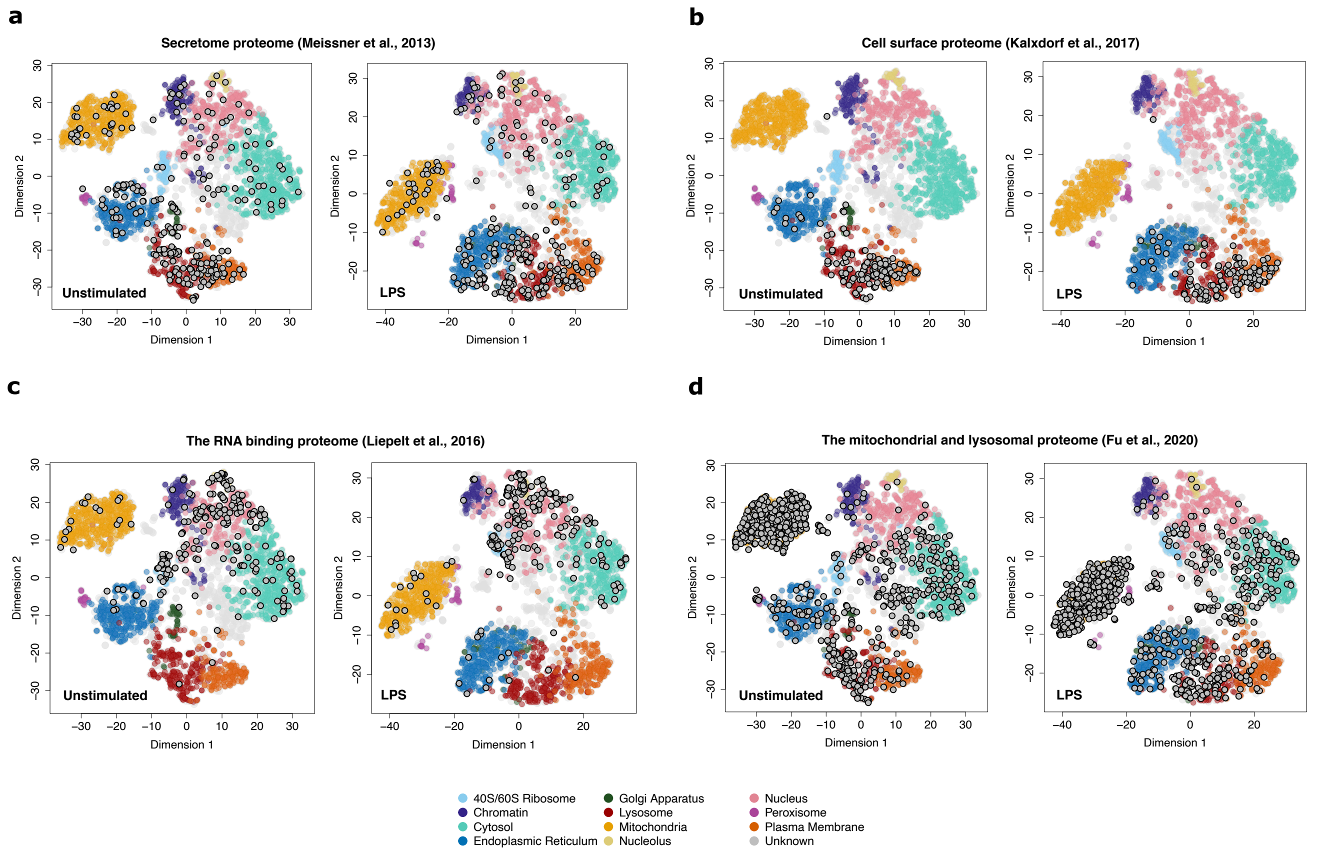

Supplementary Figure 5. The hyperLOPIT plots can be used as a scaffold to overlay proteins of interest, such as those identified by other subcellular proteomic studies. Shown are organellar proteins identified in four different studies, which have been overlaid onto the hyperLOPIT plots to validate their subcellular localisations. **a** Secretome proteome (Meissner et al., 2013), **b** cell surface proteome (Kalxdorf et al., 2017), **c** the RNA binding proteome (Liepelt et al., 2016) and **d** mitochondrial and lysosomal proteome (Fu et al., 2020).
