# Supplementary material for: Spatiotemporal proteomic profiling of the pro-inflammatory response to lipopolysaccharide in the THP-1 human leukaemia cell line
Source: Nat Commun. 2021 Oct 1;12:5773. doi: 10.1038/s41467-021-26000-9 (PMC8486773; doi:10.1038/s41467-021-26000-9)
Supplement: Supplementary file 3 — Supplementary data legends [file 41467_2021_26000_MOESM3_ESM.docx]

**Supplementary Data Legends**

**Supplementary Data 1. Temporal proteomics data.** Protein level quantitation and meta data for each replicate experiment in the LPS-stimulated time-course datasets. This includes the results from the differential expression analysis in which Limma’s paired moderated t-test using a Benjamini-Hochberg correction (1 % FDR) was used to find sets of differentially expressed proteins at each time-point. Proteins were deemed to be significantly up- or down-regulated if both their adjusted p-value ≤ 0.01 and absolute log_2_FC ≥ 0.6.

**Supplementary Data 2. Examples of some biologically related functional groups of proteins identified in the time-course analysis**. For each functional group the fold-change and Benjamini-Hochberg (1% FDR) adjusted p-values are reported for each protein generated from running Limma’s paired moderated t-test.

**Supplementary Data 3. Bayesian temporal clustering results**. **Results from the multiple dataset integration (MDI)** Bayesian temporal clustering analysis of the LPS time-course dataset.

**Supplementary Data 4. Gene Ontology (GO) annotation enrichment analysis for each of the Bayesian temporal clusters**. A Fisher’s exact test was used to determine if clusters were enriched for individual hyperLOPIT organelles (p-values were adjusted using a Benjamini-Hochberg correction).

**Supplementary Data 5. Spatial proteomics data.** Protein quantitation and meta data of the proteins identified across each set of triplicate hyperLOPIT replicates in the unstimulated (3,882 proteins) and LPS-stimulated (4,067) experiments.

**Supplementary Data 6.** **Machine learning classification results.** The results from running the TAGM-MCMC Bayesian classifier on the hyperLOPIT data. Protein quantitation and meta data of all proteins common across all six hyperLOPIT experiments found in both conditions (3,288 proteins) are shown and their associated probabilistic output from running TAGM-MCMC. A summary table displaying the distribution of organelle localisations in each condition is also shown.

**Supplementary Data 7. Protein markers.** Full list and summary table of unambiguous organelle marker proteins used for the Bayesian TAGM-MCMC classification.

**Supplementary Data 8. Translocating proteins**. The 253 proteins identified as undergoing re-localisation following 12 h of LPS stimulation within the hyperLOPIT datasets categorised by translocation type, including the final assigned organelle probability and L2 distance.

**Supplementary Data 9. LPS/TLR4 immune response and innate immune response proteins**. Of the 253 proteins identified as undergoing re-localisation following LPS stimulation, a subset of 93 were found to have been previously linked to LPS/TLR4 immune response or to innate immune response, with associated references.

**Supplementary Data 10. Proteins linked with LPS signalling by other proteomics studies.** Of the 311 proteins identified as being changed in abundance during 24 h of LPS stimulation, many have been previously linked to LPS signalling by other proteomics studies. These proteins are shown and associated references are included.

**Supplementary Data 11.** **GO enrichment of translocating proteins.** Results from the Gene Ontology (GO) annotation enrichment analysis for the 253 relocalising proteins. A Fisher’s exact test was used to determine if clusters were enriched for individual hyperLOPIT organelles (*p*-values were adjusted using a Benjamini-Hochberg correction).
