# Supplementary material for: Spatiotemporal proteomic profiling of the pro-inflammatory response to lipopolysaccharide in the THP-1 human leukaemia cell line
Source: Nat Commun. 2021 Oct 1;12:5773. doi: 10.1038/s41467-021-26000-9 (PMC8486773; doi:10.1038/s41467-021-26000-9)
Supplement: Supplementary file 15 — Reporting Summary [file 41467_2021_26000_MOESM15_ESM.pdf]

nature research

Corresponding author(s): Kathryn LIlley

Last updated by author(s): Jul 6, 2021

Reporting Summary

Nature Research wishes to improve the reproducibility of the work that we publish. This form provides structure for consistency and transparency in reporting. For further information on Nature Research policies, see our Editorial Policies and the Editorial Policy Checklist.

Statistics

For all statistical analyses, confirm that the following items are present in the figure legend, table legend, main text, or Methods section.

n/a	Confirmed	
☐	☒	The exact sample size (n) for each experimental group/condition, given as a discrete number and unit of measurement
☐	☒	A statement on whether measurements were taken from distinct samples or whether the same sample was measured repeatedly
☐	☒	The statistical test(s) used AND whether they are one- or two-sided<br>*Only common tests should be described solely by name; describe more complex techniques in the Methods section.*
☒	☐	A description of all covariates tested
☐	☒	A description of any assumptions or corrections, such as tests of normality and adjustment for multiple comparisons
☐	☒	A full description of the statistical parameters including central tendency (e.g. means) or other basic estimates (e.g. regression coefficient) AND variation (e.g. standard deviation) or associated estimates of uncertainty (e.g. confidence intervals)
☐	☒	For null hypothesis testing, the test statistic (e.g. F, t, r) with confidence intervals, effect sizes, degrees of freedom and P value noted<br>*Give P values as exact values whenever suitable.*
☐	☒	For Bayesian analysis, information on the choice of priors and Markov chain Monte Carlo settings
☒	☐	For hierarchical and complex designs, identification of the appropriate level for tests and full reporting of outcomes
☒	☐	Estimates of effect sizes (e.g. Cohen's d, Pearson's r), indicating how they were calculated

Our web collection on statistics for biologists contains articles on many of the points above.

Software and code

Policy information about availability of computer code

Data collection

• LPS time-course data were acquired on an Orbitrap Q-Exactive™ MS (Thermo Scientific)
• The hyperLOPIT data was acquired on the Orbitrap™ Fusion™ Lumos™ Tribrid™ instrument (Thermo Scientific)
• Both instruments were coupled to Dionex Ultimate 3000 RSLCnano systems (Thermo Scientific)
• The acquisition for all experiments was performed in XCalibur v3.0.63 (Thermo Fisher Scientific)
• The output proteomics .raw files generated from XCalibur were processed with Proteome Discoverer v2.1 (Thermo Fisher Scientific) and Mascot server v2.3.02 (Matrix Science).
• ELISA plates were read on a CLARIOstar microplate reader (BMG LABTECH)
• Western blots were visualised using a myECL Imager (Thermo).
• Immunofluorescent images were captured using a Leica SP5 confocal microscope at 63 x magnification.

Data analysis

• The hyperLOPIT and timecourse data analysis was largely analysed using the statistical programming language R (v3.5.1) [http://www.R-project.org/] with the open-source open-development R Bioconductor (v3.7) packages MSnbase (v2.8.3) and pRoloc (v1.23.2). MSnbase provides a platform for manipulation and processing of mass spectrometry data and the pRoloc package implements machine learning and visualisation methods for the analysis of quantitiative proteomics data.
• The freely available Database for Annotation, Visualisation and Integrated Discovery software (DAVID v6.8; https://david.ncifcrf.gov/) was used for Gene Ontology enrichment analysis. The DAVID web-server provides a platform fo the mining of gene lists to identifying enriched biological themes.
• The open-source MDI-GPU software (v 1.0 (https://warwick.ac.uk/fac/cross_fac/zeeman_institute/zeeman_research/software v1 July 2015)) was used to perform integrative Bayesian model-based clustering.
• TAGM-MCMC in pRoloc (v1.23.2) was used for machine learning protein classification.
• The MARS Data Analysis Software (BMG LABTECH) and Prism V8 were used for ELISA data analysis.

- The Fiji Software was used for image processing.
- All R code used for data analysis and generation of figures is openly and freely available https://github.com/CambridgeCentreForProteomics/thp-lopit-2021 along with a vignette at https://cambridgecentreforproteomics.github.io/thp-lopit-2021/.

For manuscripts utilizing custom algorithms or software that are central to the research but not yet described in published literature, software must be made available to editors and reviewers. We strongly encourage code deposition in a community repository (e.g. GitHub). See the Nature Research guidelines for submitting code & software for further information.

Data

Policy information about availability of data

All manuscripts must include a data availability statement. This statement should provide the following information, where applicable:

- Accession codes, unique identifiers, or web links for publicly available datasets
- A list of figures that have associated raw data
- A description of any restrictions on data availability

Raw data are available at the PRIDE proteomics repository dataset identifier PXD023509 (Username: reviewer_pxd023509@ebi.ac.uk ; Password: RmZ2XGJA) and the processed protein and peptide level data is also freely and openly available in the R Bioconductor pRolocdata package (≥v1.27.3), as well as in Supporting Table 1 (timecourse data), Supporting Table 5 (hyperLOPIT data) of this manuscript.
The spatial datasets can also be viewed, mined and interrogated via the accompanying R Shiny application which is available for the community at http://proteome.shinyapps.io/thp-lopit/. Figures 2 - 7 and Supplementary Figures 1 - 5 are all generated directly from the processed raw data (as detailed in https://cambridgecentreforproteomics.github.io/thp-lopit-2021/).

Field-specific reporting

Please select the one below that is the best fit for your research. If you are not sure, read the appropriate sections before making your selection.

☒ Life sciences ☐ Behavioural & social sciences ☐ Ecological, evolutionary & environmental sciences

For a reference copy of the document with all sections, see nature.com/documents/nr-reporting-summary-flat.pdf

Life sciences study design

All studies must disclose on these points even when the disclosure is negative.

Sample size	three biological replicates were used for each time point for the temporal proteomics study and for each time point used for spatial proteomics experiments. Three biological replicates were used for cytokine secretion. Western blots were run once using a lysate from each time point.
Data exclusions	One TMT channel was removed from the final hyperLOPIT analysis in both the unstimulated and stimulated conditions, due to erroneous labelling of insoluble material during the sample preparation for one replicate
Replication	three biological replicates for each condition were used and all replicates were successful
Randomization	Confounding was controlled by blocking rather than randomization
Blinding	No were patients involved in this study and experimentalists need to know the conditions to interpret the data

Reporting for specific materials, systems and methods

We require information from authors about some types of materials, experimental systems and methods used in many studies. Here, indicate whether each material, system or method listed is relevant to your study. If you are not sure if a list item applies to your research, read the appropriate section before selecting a response.

Materials & experimental systems

n/a	Involved in the study	
☐	☒	Antibodies
☐	☒	Eukaryotic cell lines
☒	☐	Palaeontology and archaeology
☒	☐	Animals and other organisms
☒	☐	Human research participants
☒	☐	Clinical data
☒	☐	Dual use research of concern

Methods

n/a	Involved in the study	
☒	☐	ChIP-seq
☒	☐	Flow cytometry
☒	☐	MRI-based neuroimaging

Antibodies

Antibodies used	Polyclonal Goat Anti-Rabbit HRP, DAKO,(1:5000 Cat #: P0448)<br>Polyclonal Goat Anti-Mouse HRP, DAKO, (1:5000Cat #: P0447)<br>Goat anti-Rabbit IgG (H+L) Cross-Adsorbed Secondary Antibody, Alexa Fluor 488, Thermo, Cat #: A11008<br>rabbit anti-p62(1:2000; Cell Signaling, Cat #: 5114), aka SQSTM1<br>mouse anti-CLEC11a (1:1000; R&D Systems, Cat #:MAB1904),<br>rabbit anti-cdc42 (1:500; Proteintech, Cat #: 10155-1-AP),<br>rabbit anti-IL-1β(1:5000; Cell Signaling, Cat #: 12703),<br>rabbit anti-actin (1:5000; Sigma, Cat #: A2066)
Validation	rabbit anti-p62(1:2000; Cell Signaling, Cat #: 5114) -Validated by the manufacturer and in literature for western blotting. https://www.cellsignal.co.uk/products/primary-antibodies/sqstm1-p62-antibody/5114. This product has met all of the quality control standards defined by Cell Signaling Technology, Inc. Lot #: 6.<br>mouse anti-CLEC11a (1:1000; R&D Systems, Cat #:MAB1904), Lot Number#: KXY021906A.Tested by the manufacturer using ELISA and western blot. https://www.rndsystems.com/products/human-scgf-clec11a-antibody-239029_mab1904. REF that uses the antibody for WB - https://www.ncbi.nlm.nih.gov/pmc/articles/PMC3192683/<br>rabbit anti-cdc42 (1:500; Proteintech, Cat #: 10155-1-AP), Validated by the manufacturer and in literature for use in immunofluorescence and western blotting. https://www.ptglab.com/products/CDC42-Antibody-10155-1-AP.htm#product-information. Lot #: 1<br>rabbit anti-IL-1β(1:5000; Cell Signaling, Cat #: 12703), Validated by the manufacturer and in literature for western blotting. https://www.cellsignal.co.uk/products/primary-antibodies/il-1b-d3u3e-rabbit-mab/12703. Lot #: 1<br>rabbit anti-actin (1:5000; Sigma, Cat #: A2066) Validated by the manufacturer and in literature for western blotting. https://www.sigmaaldrich.com/GB/en/product/sigma/a2066. Lot #: 019M4777V<br>Polyclonal Goat Anti-Rabbit HRP, DAKO, Cat #: P0448, Lot #: 20023997<br>Polyclonal Goat Anti-Mouse HRP, DAKO, Cat #: P0447, Lot #: 20062696<br>Goat anti-Rabbit IgG (H+L) Cross-Adsorbed Secondary Antibody, Alexa Fluor 488, Thermo, Cat #: A11008, Lot #: 913909<br>Alexa Fluor 546 Phalloidin, Thermo, Cat #: A22283, Lot #: 1775962<br>Manufacturers verified the specificity of all the secondary antibodies.

Eukaryotic cell lines

Policy information about cell lines

Cell line source(s)	THP-1 cells (ATCC® TIB-202™)
Authentication	The cells were purchased from ATCC. No validation technique was used
Mycoplasma contamination	the cells tested negative for mycoplasma
Commonly misidentified lines<br>(See ICLAC register)	The ICLAC site - version 11, released 8th June 2021 was checked and no commonly misidentified cell lines were used in the study.
